# Growth Type and Functional Trajectories: An Empirical Study of Urban Expansion in Nanjing, China

**DOI:** 10.1371/journal.pone.0148389

**Published:** 2016-02-04

**Authors:** Jianglong Chen, Jinlong Gao, Feng Yuan

**Affiliations:** 1 Key Laboratory of Watershed Geographic Sciences, Nanjing Institute of Geography and Limnology, Chinese Academy of Sciences, Nanjing 210008, China; 2 University of Chinese Academy of Sciences, Beijing 100049, China; Peking UIniversity, CHINA

## Abstract

Drawing upon the Landsat satellite images of Nanjing from 1985, 1995, 2001, 2007, and 2013, this paper integrates the convex hull analysis and common edge analysis at double scales, and develops a comprehensive matrix analysis to distinguish the different types of urban land expansion. The results show that Nanjing experienced rapid urban expansion, dominated by a mix of residential and manufacturing land from 1985 to 2013, which in turn has promoted Nanjing’s shift from a compact mononuclear city to a polycentric one. Spatial patterns of three specific types of growth, namely infilling, extension, and enclave were quite different in four consecutive periods. These patterns result primarily from the existing topographic constraints, as well as government-oriented urban planning and policies. By intersecting the function maps, we also reveal the functional evolution of newly-developed urban land. Moreover, both self-enhancing and mutual promotion of the newly developed functions are surveyed over the last decade. Our study confirms that the integration of a multi-scale method and multi-perspective analysis, such as the spatiotemporal patterns and functional evolution, helps us to better understand the rapid urban growth in China.

## Introduction

Over the past several decades, unprecedented urbanization, characterized by a demographic shift from rural to urban areas and urban land expansion, has taken place on a global level. Between 1970 and 2011, the number of people dwelling in cities has increased from 1.35 billion to 3.63 billion, witnessing an increase of 169% [1]. At the same time, urban land expansion has been even more dramatic [2]. Globally, urban areas quadrupled between 1970 and 2000 [3], which is reportedly an expansion, on average, of twice the urban population growth rates in recent years [4]. As the most fundamental component of global environmental change [5], the urban expansion phenomenon has wide-ranging significance in terms of ecosystem functioning and services on both local and global scales [6–8], with equally far-reaching consequences for human wellbeing [9]. To date, while a large amount of literature has considered the changing patterns [10–13], driving dynamics [14–18], potential implications [6, 19], and the spatial modeling [20, 21] of the changes in land use brought about by urbanization, this type of urban growth is still relatively less understood, and the trajectories of the functional evolution of newly-expanded urban land remain largely unexplored.

With potentially the greatest human resettlement experiment in history, China’s urbanization has attracted considerable scholarly attention, particularly the dramatic urban expansion and related challenges to achieving sustainable development [22, 23]. In a step designed to address these challenges and towards achieving sustainable development, the Chinese government also introduced the concept of ‘New Urbanization’ in March, 2014, under the auspices of the ‘National New-type Urbanization Plan’ (NUP). The NUP covers almost every aspect of urbanization, from rural-urban integration to sustainable development [24]. As one of the three policy challenges specified in the NUP, urban land expansion plays an increasing and important role in China’s dream of sustainable development [25]. Given such political demands, Chinese researchers must re-learn and understand the concept of the spatial development of urban land, as well as its functional evolution trajectories over the past few decades.

The spatial pattern of an urban region is a consequence of the interaction between various types of driving forces, including the natural and socioeconomic factors [26]. Along with these, the spatial heterogeneity of factors (such as topography, traffic accessibility, population and market conditions) could further influence urban morphology and cause different typologies of urban growth [27, 28]. That a rich body of literature has been produced to explain the different types of urban growth does not necessarily mean that the issue has been sufficiently resolved, for at least two key reasons. On the one hand, from the prospective of typology, Forman [29] divided urban growth into three types: infilling, extension, and enclave. For identifying and quantifying urban growth types, Xu; Liu; Zhang; An; Yu and Chen [27] and Liu; Li; Chen; Tan; Li and Ai [30] proposed a common edge analysis and Landscape Expansion Index (LEI) with buffer zone analysis, respectively. However, these identification methods can only distinguish the growth types at a patch scale, which might be misleading at larger city or regional levels [31]. For instance, one expansion patch in the inner city proper will be identified as enclave growth, provided that patch has no common edge with the pre-growth existing patches, though in practice, this growth should be scientific infilling. On the other hand, to avoid misidentifying the different types of expansion, Liu; Wang; Zhuang; Zhang and Hu [32] developed a new method based on the principle of convex hull, which is used to define growth types by the proportion of the area located inside or outside the convex hull of a city’s outline. However, these scholars only identified the infilling and extension growth on the macro-city scale. Faced with the problem of lacking a comprehensive quantitative approach to identify the different types of urban land expansion at multi-scale level, we have developed a new approach, which has rarely been applied in previous studies. Our method is to employ a comprehensive matrix analysis by integrating the methods of two different spatial scales. We also linked the different types of growth with different land use functions, which might be the fundamental driving mechanism of urban land expansion. In particular, the evolutionary process of urban land use function is detected and explained in this study.

In this study, we combined the two analysis methods of common edge and convex hull to investigate the different types of urban growth in Nanjing, China, as well as its functional evolution characteristics. Furthermore, we proposed two simple methods which can be employed to distinguish urban growth typology. Then, the quantitative composition and distribution of the different growth types were analyzed during different periods. Finally, the functional evolution characteristics of the newly-grown patches are comprehensively discussed. The process also takes local reinforcement into consideration. The purpose of this paper is to address the following questions: 1) How does an urban area grow over time? 2) What are the functions of the newly-grown areas?

## Data and Methodology

### Study area and data preprocessing

**Fig 1:**
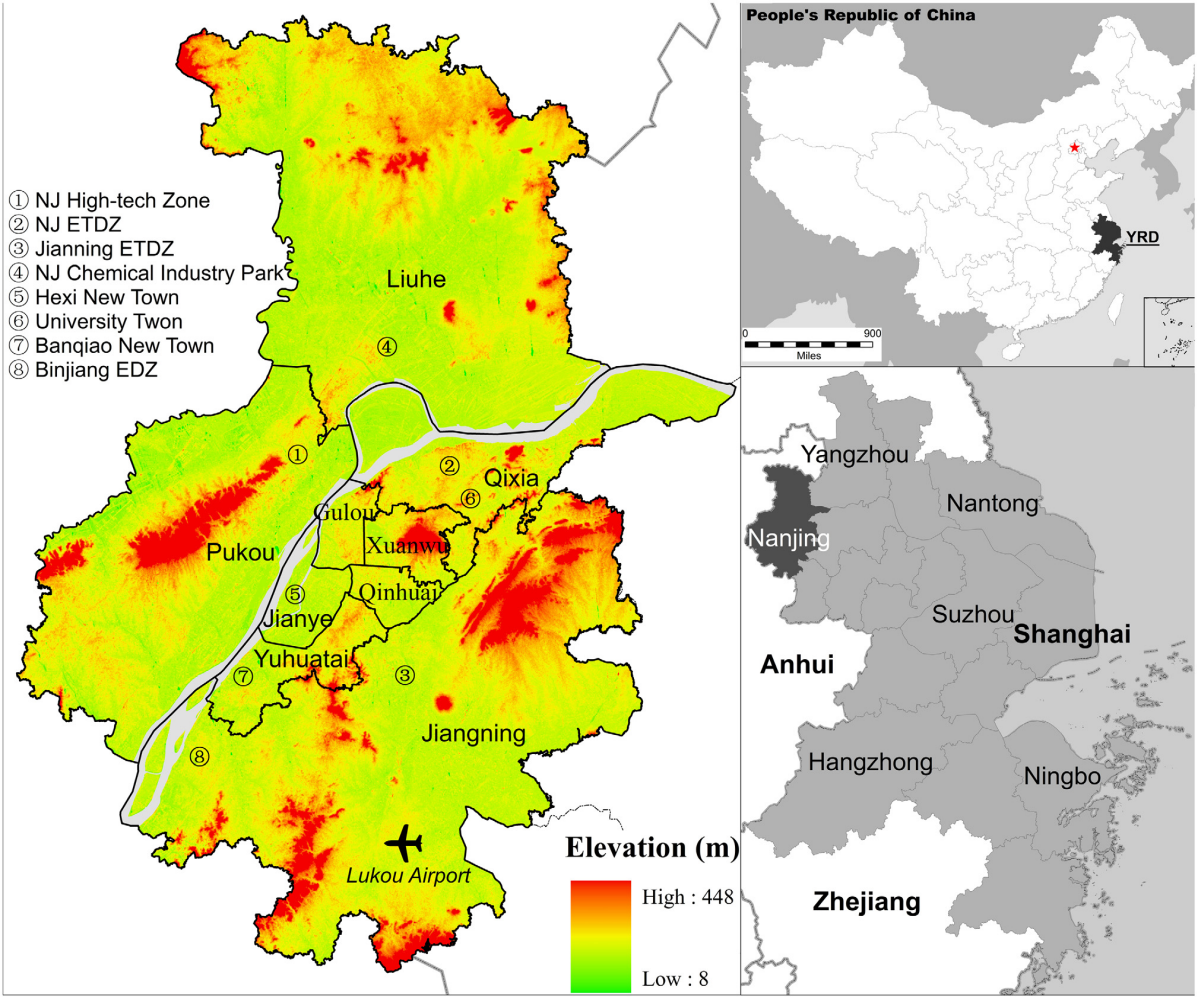
The location and administrative division of the study area.

Nanjing includes the city proper (four districts) and five suburban districts, with a total land area of 4,723 sq.km. Approximately 15 percent of the total area is occupied by low hills. As one of the three core cities in the Yangtze Delta (Fig 1) and the world’s sixth-largest economic center, Nanjing is a classic representative of the rapidly growing and globalizing cities which are at the interface of globalization, national reforms, and bottom-up development. With a total population of 8.16 million and an urban population of 6.55 million, Nanjing is the second-largest commercial center in the East China region, after Shanghai (NSB, 2014). The total area of land under construction in 2013 was 1,519 sq.km, accounting for no less than 83% of the entire municipality.

**Fig 2:**
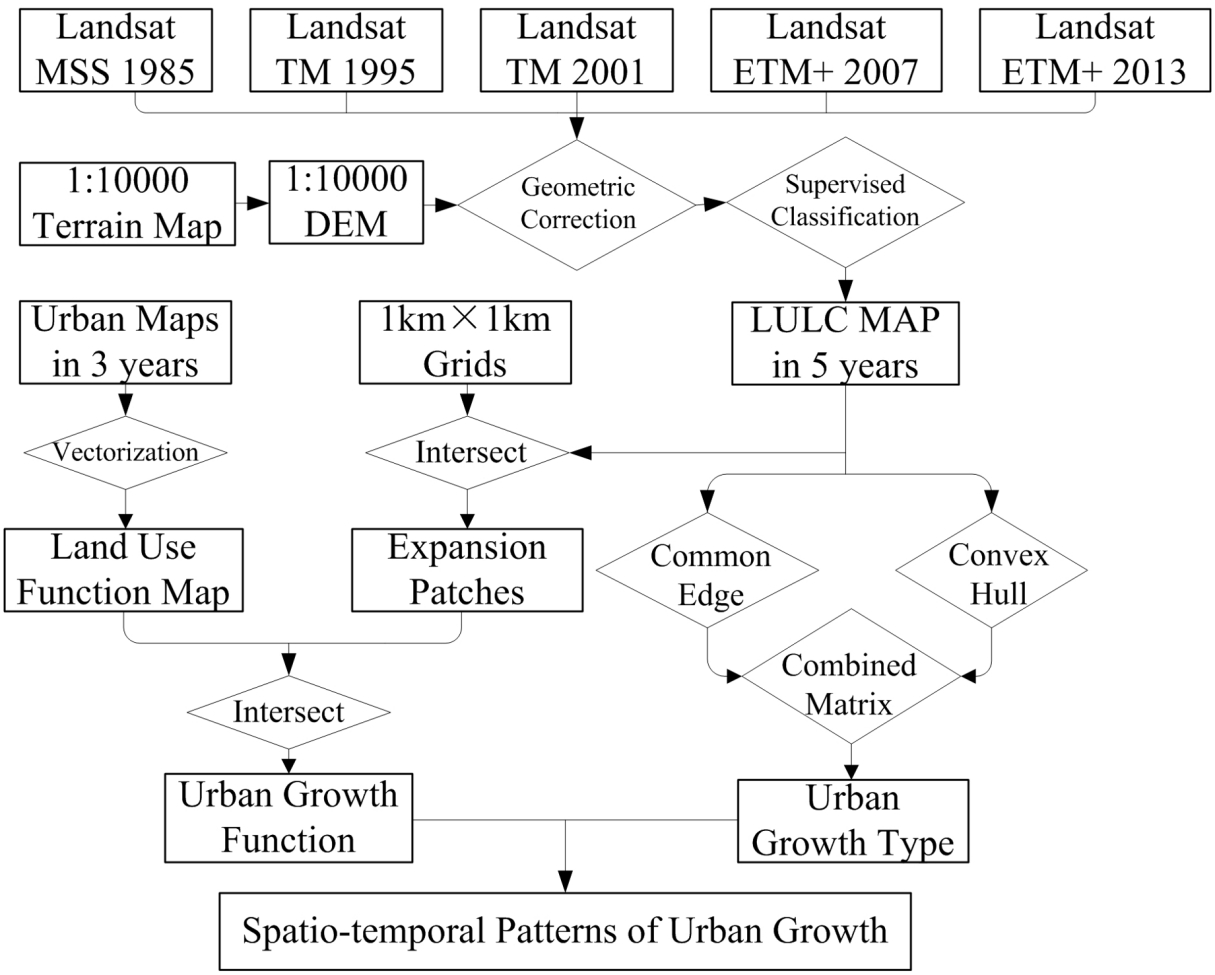
Flowchart of detection procedures.

Our data is based mainly on information received from five cloud-free Landsat Multispectral Scanners (MSS), Thematic Mappers (TM), and Enhanced Thematic Mapper-plus (ETM+) images from May, 1985, July, 1995, May, 2001, August 2007, and August, 2013 (Path: 120, Row: 38). We processed the data in the following ways (Fig 2): First, we derived layers of land-use in different years from Landsat images by using a hybrid of supervised classification and manual interpretation in ERDAS Imagine software, with the kappa coefficient over 85% for a land-use map over five years. Five broad, land cover types (i.e., urban land, cropland, forest, water, and natural cover) were classified based on these images, using the Maximum Likelihood method. We further merged the other four types as non-urban areas, based on the classifications of the Second National Land Survey, because we only focused on urban land in this study.

Meanwhile, we intersected the study area with a fishnet grids of 1square kilometer, and we identified the land use function based on the vectored maps of land use functions in 2001, 2007, and 2013. Then, the functions of the newly-developed patches were detected by intersecting the expansion patches and the function maps. After distinguishing three growth types, the expansion trajectory of urban land expansion was discussed from the perspectives of types and functions.

### Urban growth types identified with common edge

Xu; Liu; Zhang; An; Yu and Chen [27] developed the Urban Growth Type Index (UGTI) to define the three aforementioned urban growth types. Specifically, the identifying process was accomplished using professional spatial analysis software ArcGIS, version 9.3. Firstly, we intersected the newly expanded patches and pre-growth areas to get the common edge [33]. We then calculate the UGTI using the following equation:

$$UGTI=L_{common}/P_{new}$$ (1)

where *P*_*new*_ represents the perimeter of a newly-grown urban patch, *L*_*common*_ is the length of the common edge of this newly-grown urban patch and existing pre-growth urban patch or patches, and *UGTI* is the urban growth type index, ranging from 0 to 1. As illustrated in Gao and others’ study [33], the urban growth type is identified as infilling when *UGTI* > 0.5, as an extension when 0 < *UGTI* ≤ 0.5, and as an enclave when *UGTI* = 0 which indicates no common edge.

### Urban growth types identified with convex hull

Though the UGTI can be used to identify the growth types at a patch scale, the results are not as convincing at more macro-city or regional scales, as mentioned above. Thus, another quantitative method to distinguish the three growth types was proposed using Desktop 2013 of the Feature Manipulate Engine (FME), a unique data integration platform developed by Safe Software. Firstly, we created a point feature class based on the boundary of the existing pre-growth urban areas. Secondly, we derived the minimal external convex polygon, namely the convex hull of existing urban areas, using the Hull Accumulator function under the Workbench platform of FME. Lastly, we identified the growth types based on the percent of newly-grown land located in the inner portion of the hull. According to Gao, Chen, Yuan, Wei and Chen [33], an urban growth type is defined as infilling if the inner segment is larger, and vice versa extension. If the newly expanded area is completely outside of the convex hull, that area is designated as an enclave.

### Completed urban growth types based on combined matrix

Using the two methods mentioned above, we can easily get two growth types for every newly-grown patch, though the two types might be different for some patches. To coordinate any possible inconsistencies, we designed a matrix rule (Table 1). Based on this matrix, we reidentified the growth type when the two results are different. If one patch is identified as infilling growth based on the convex hull method, it will never be an enclave in practice and should have been an extension when it is enclave in the common edge method. Similarly, if one area is identified as an enclave growth area based on the convex hull method, it is an enclave no matter what type it is in the common edge method. For the extension growth area based on the convex hull method, the infilling growth area based on the common edge method should be reclassified as an extension.

**Table 1:** Rules of Matrix-based analysis of urban growth types.

		**Common edge**		
		**Infilling**	**Extension**	**Enclave**
**Convex hull**	**Infilling**	Infilling	Extension	Extension
	**Extension**	Extension	Extension	Enclave
	**Enclave**	Enclave	Enclave	Enclave

Note: See the visual illustrations to describe all possible scenarios in S1 Fig.

## Spatiotemporal Patterns of Three Growth Types

### Dynamics of urban growth

**Fig 3:**
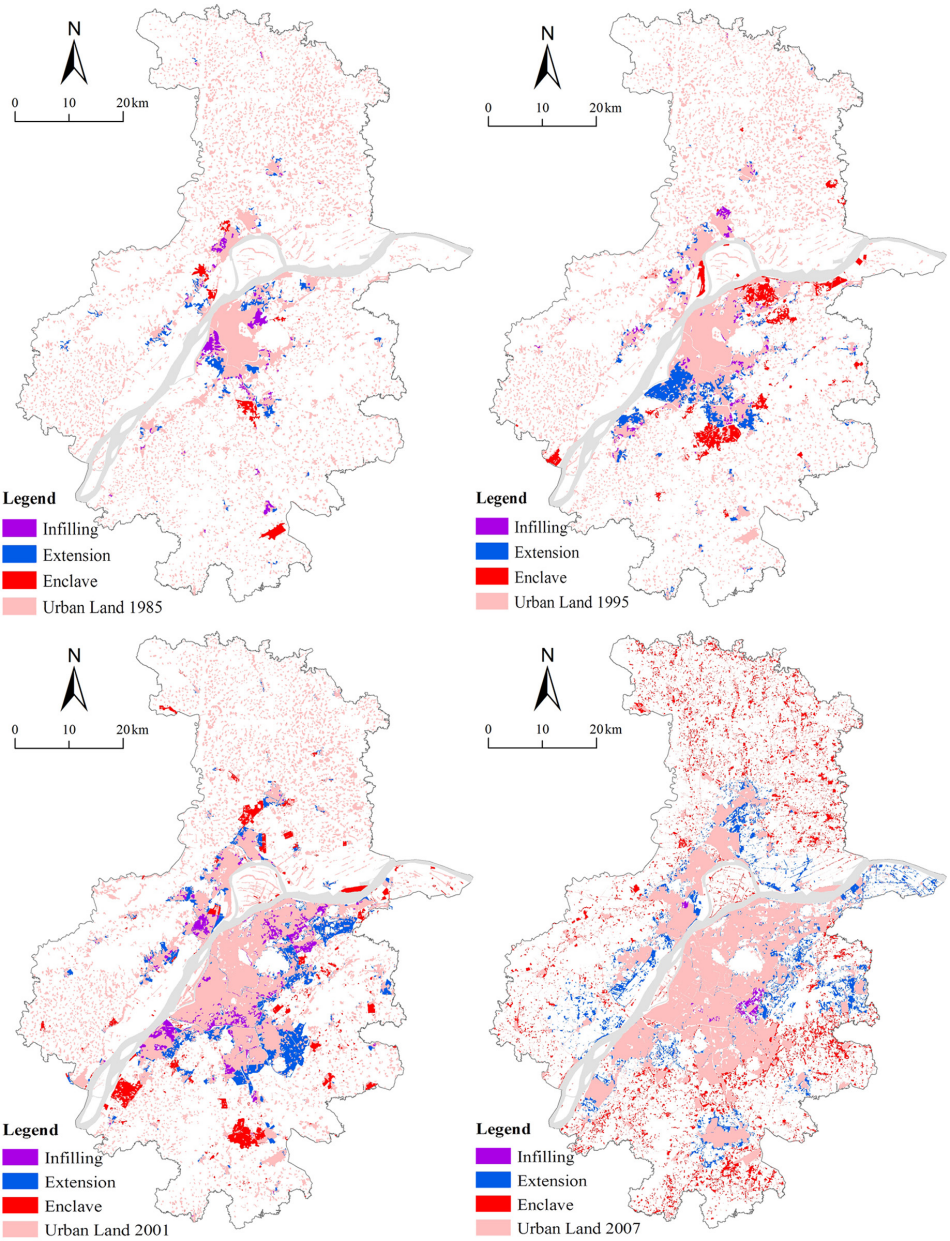
Patterns of different types of urban growth in Nanjing from 1985 to 2013.

Over the past two decades, Nanjing had undergone rapid urbanization, which can be seen from the continuous expansion of its urban land (Fig 3). The total urban land area increased from 647.1 sq.km in 1985, to 1864.4 sq.km in 2013, representing an annual growth rate of 3.85% during the study period (Table 2). Spatially, urban land centered in the old city proper, as well as in the original Dachang (the present-day Liuhe) district in the north of the Yangtze River Delta, and Jiangning in the south of the area proper in 1985. It gradually spread to the northeast and southwest of the area proper over the following ten years (1985–1995). During this relatively stable period, the amount of urban land also increased, surrounding the two sub-centers of Jiangbei and Jiangning. All this patterns of growth were closely linked with the wave of “zone fever” demonstrated by the establishment of the three national level development zones (DZs), including the Nanjing High-tech Zone, the Nanjing Economic Technology Development Zone (ETDZ), and the Jiangning ETDZ. In addition, “project fever” was clearly demonstrated by the introduction of Lukou International Airport [34]. The development of DZs largely stimulated the local governments’ enthusiasm for growth and led to the establishment of three new districts, namely Jiangbei in the north, Xianlin in the northeast, and Jiangning in the south. Consequently, the development of urban land sharply increased in these zones during the period from 1995 to 2001. Meanwhile, the metropolitan government began building the Hexi New Town to the southwest of the city center, starting in 2001, which also led to an apparent growth in urban land there. This growth was consistent with Luo and Wei [35] study of the probability surface of urban growth in Nanjing.

**Table 2:** Urban land expansion in Nanjing, 1985–2013.

Year	Total urban land (sq. km)	Period	Annual growth rate (%)	Annual expansion (sq. km)
**1985**	647.1	1985–1995	1.48	10.24
**1995**	749.5	1995–2001	5.37	46.04
**2001**	1025.7	2001–2007	5.42	63.66
**2007**	1407.7	2007–2013	4.79	76.11
**2013**	1864.4	1985–2013	3.85	43.47

At the onset of the 21^st^ century, urban land expansion further expedited due to the aforementioned “one town and three districts” spatial planning process. In addition to these inner suburban expansions, urban land also increased rapidly to the southwest, along the Yangtze River, where there were industrial bases, and to the south of the Jiangning New District close to Lukou International Airport. In the six years leading up to 2013, urban land expansion accelerated even further, and mainly occurred in Jiangbei, surrounding the Nanjing Chemical Industrial Park and Nanjing High-tech Zone, as well as areas along the Yangtze River, coinciding with the initiation of the “Great Leap Forward of Jiangbei”. Coincidentally, satellite towns in the east and south of Jiangning also recently witnessed rapid urban land expansion, thereby indicating the agglomerations of activities in the sub-centers.

To summarize (Fig 3 and Table 2), urban expansion in Nanjing was relatively slow and was around the old city center prior to 1995, which indicated that Nanjing was once a compact mononuclear city [35]. Since the mid-1990s, however, the trend towards urban expansion accelerated, and the polycentric structure became clearer like the FDI-fuelled cities, such as Hangzhou [36] and Guangzhou [37]. With the rapid growth of urban land in the inner suburban areas, Nanjing experienced rapid polycentric urban development over the following years. In brief, recent expansion in satellite towns and the outer suburban areas has effectively made the sprawl of built-up areas pancake together. While such patterns of development may promote Nanjing’s integration with surrounding cities (e.g. Zhenjiang and Yangzhou), it can also be a great challenge for sustainable urban development.

### Patterns of the common edge based growth types

To further understand the detailed spatial patterns of urban land expansion, three urban growth types (i.e., infilling, extension and enclave) were identified for the newly-developed urban patches, based on the common edge analysis method (Table 3). From 1985 to 1995, infilling growth in the old city proper dominated the expansion of Nanjing, which necessarily resulted in a compact mononuclear city. Extension growth was distributed sparsely in three subcenters, especially in Jiangning, which was an independent county in terms of administration. Enclave growth occupied only 2% of the total newly-developed urban land in the city proper and three sub-centers combined. In the second period (from 1995 to 2001), the formation of the basic urban structure extension was gradually replaced by infilling as the primary growth type. Particularly, the proportions of infilling growth in Jiangning and Xianlin increased by 26.21% and 15.35% respectively, in a ten-year period. Thus, subcenters developed as officials had planned.

**Table 3:** Areas of different urban growth types based on common edge analysis.

	1985–1995		1995–2001		2001–2007		2007–2013	
	Area (sq.km)	% of Total	Area (sq.km)	% of Total	Area (sq.km)	% of Total	Area (sq.km)	% of Total
**City proper**	**29.53**	**49.68**	**25.78**	**25.59**	**16.36**	**17.01**	**3.37**	**5.47**
Infilling	18.28	30.75	19.50	19.36	12.10	12.58	0.04	0.06
Extension	10.91	18.35	6.23	6.19	4.21	4.38	2.88	4.69
Enclave	0.35	0.58	0.05	0.05	0.05	0.05	0.45	0.73
**Sub-JB**	**13.89**	**23.37**	**16.91**	**16.78**	**23.79**	**24.74**	**37.69**	**61.22**
Infilling	5.29	8.90	7.05	6.99	5.87	6.10	0.02	0.03
Extension	8.43	14.18	9.64	9.57	17.26	17.95	31.42	51.04
Enclave	0.17	0.29	0.22	0.22	0.66	0.69	6.25	10.15
**Sub-XL**	**4.47**	**7.52**	**23.98**	**23.80**	**21.43**	**22.28**	**11.31**	**18.38**
Infilling	0.10	0.16	15.62	15.51	19.69	20.48	0.12	0.20
Extension	3.79	6.38	7.37	7.32	1.72	1.79	9.73	15.81
Enclave	0.59	0.99	0.98	0.98	0.01	0.01	1.46	2.37
**Sub-JN**	**11.55**	**19.42**	**34.08**	**33.82**	**34.59**	**35.97**	**9.19**	**14.92**
Infilling	0.94	1.58	27.99	27.78	14.04	14.60	0.01	0.02
Extension	10.61	17.85	5.75	5.71	20.52	21.33	8.60	13.97
Enclave	0.00	0.00	0.33	0.33	0.03	0.03	0.57	0.93
**Total**	**59.44**	**100**	**100.75**	**100**	**96.17**	**100**	**61.56**	**100**

Between 2001 and 2007, the percentage of extension growth increased to almost 45.45%, which was very close to the infilling growth rate of 53.76%. The most obvious increase occurred in Jiangning and the Jiangbei sub-centers, where the urbanization process accelerated under the guidance of the third round urban master plan of sub-center development [38]. In contrast, the enclave growth rate decreased to approximately 0.78%. All these facts manifested the maturity and compact trend of the built-up areas. Thereafter, the infilling growth rate almost disappeared in all four regions in the period from 2007 to 2013. In addition, the extension growth accounted for nearly 90% of all expansion, reflecting the concentrated urban form of Nanjing and the maturity and development of the subcenters.

Another interesting finding is that the ranks of the expansion areas in the city proper and three subcenters constantly changed throughout the entire study period. In the first period (from 1985 to 1995), expansion mainly occurred in the city proper, followed by Jiangbei, Jiangning, and Xianlin, which once again confirmed the mononuclear pattern of Nanjing. In the following two periods (from 1995 to 2001 and 2001 to 2007), land expansion in the city proper fell to second and fourth place, respectively, whereas the expansion in Jiangning leapt to first place. When the strategic core of urban development shifted to the north of the Yangtze River, Jiangbei ranked first, followed by Xianlin. Both surpassed Jiangning in the years from 2007 to 2013. Though the expansion ranks of city centers and subcenters changed greatly during the study periods, it should be noted that subcenters didn’t experience rapid development until the city proper grew more compact. Also, with the shift of urban growth from the main center to the subcenters, the agglomeration of activities in the subcenters, and the official planning, polycentric development was finally shaped under the guidance of official planning.

### Patterns of the combined growth types

Additional macro-level analysis of urban growth types was carried out by combining the common edge and convex hull methods over different periods (Fig 3). Overall, patterns of growth types based on the combined matrix analysis are similar with the common edge-based patterns, though detailed differences exist. In particular, the proportion of enclave growth increased enormously. From 1985 to 1995, most of the infilling growth occurred in the city proper and original county centers. The establishment of the Nanjing High-tech Zone, the Jiangning ETDZ, and Lukou International Airport (see in Fig 1) promoted the enclave growth of urban land both northwards and southwards. Extension growth was initially concentrated along the river. With the construction of Hexi New Town at the southwest of the city proper and with the continuous development of the Jianging ETDZ in the following six years (from 1995 to 2001), the extension growth later spread outwards. During the same period of 1995–2001, the government planned the construction of Xianlin University Town at the northeast of the city proper. The development of the Nanjing ETDZ and the Jiangning ETDZ further accelerated. Consequently, large scale enclave growth occurred in these areas with a relative low density [38].

However, due to two significant administrative division adjustments for the city proper in 2002 [39], the government-oriented strategies of “crossing the Yangtze River and developing northward” was implemented. Meanwhile, the city launched a series of new policies to promote economic reform and growth, including the 2001 Nanjing Master Plan, which presented ‘‘one city and three districts” as the desired urban structure [40]. As a result, the rapid development of subcenters led to a great deal of enclave growth of urban land northward, southward, and along the river. Extension growth was not only concentrated around the original built-up areas, but also expanded from the previous enclave areas of 2001 to 2007. In the last six years of the study period, the dominant growth type was extension, surrounding the existing functional zones including three sub-centers and the various levels of DZs. Furthermore, while infilling growth occurred at the east of the city proper, enclave growth was witnessed near the remote satellite towns. In total, infilling, extension, and enclave growth occurred from inner to suburban areas and showed a concentric distribution pattern, which is consistent with the findings of Xu; Liu; Zhang; An; Yu and Chen [27].

## Functional Characteristics of Three Growth Types

### Functional evolution of newly developed patches

To shed further light on the heterogeneity and dynamics of three growth types, we also detected the functional characteristics of the newly-developed urban land. To ensure consistency and to simplify the processing procedures, data processing was accomplished using fishnet grids of 1 square kilometer, as shown in Fig 2, while grids with less than 10 percent expansion were eliminated. Finally, we obtained 1,414 expansion grids with relatively accurate functions. The number of grids obtained for each period was 334 for 1995 to 2001, 538 for 2001 to 2007, and 542 for 2007 to 2013, representing 84%, 82% and 79% of the total expansion, respectively.

**Fig 4:**
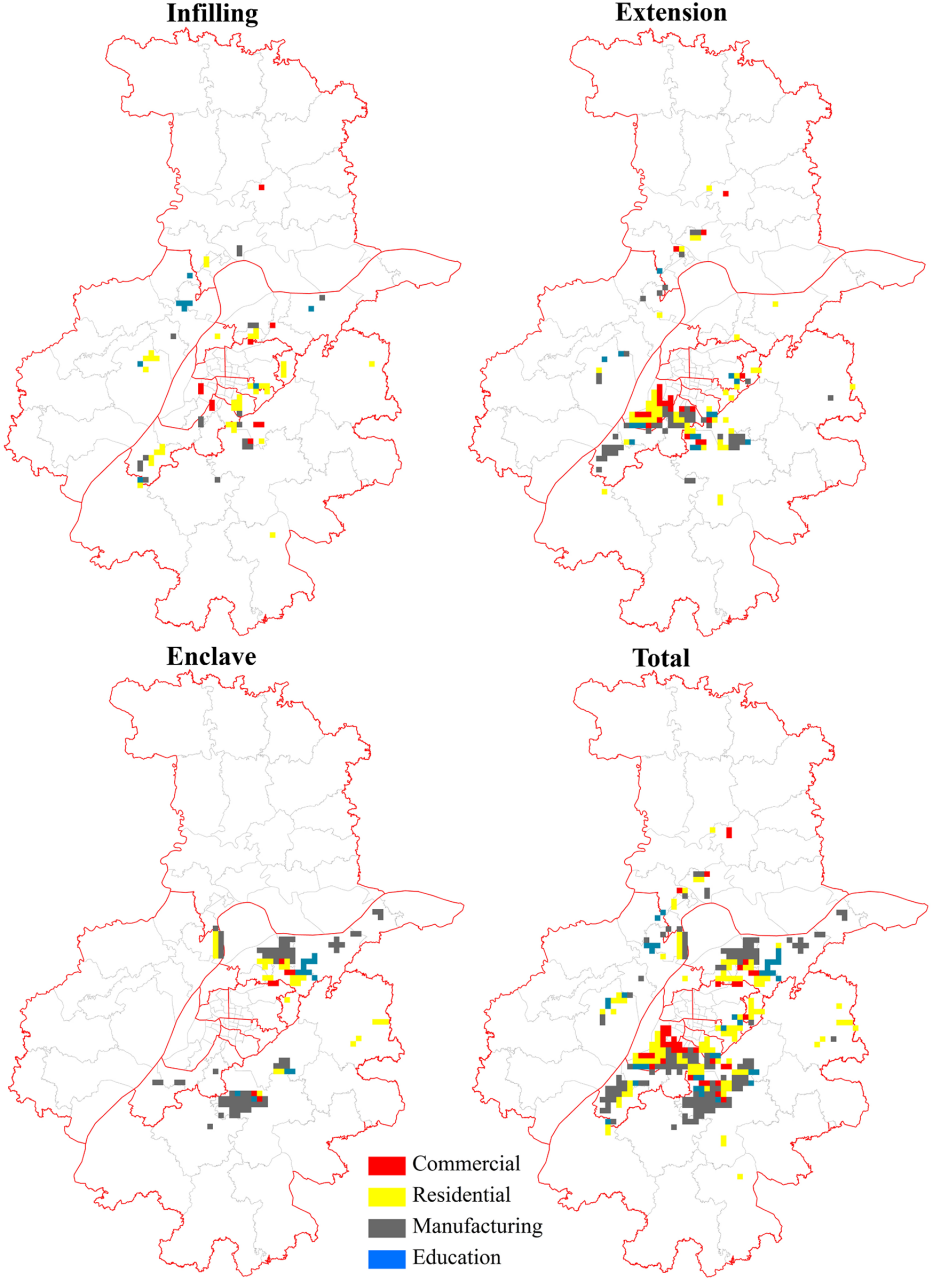
Main functions of newly grown urban patches from 1995 to 2001.

Urbanization process was accelerated in Nanjing, during the period from 1995 to 2001. The major infilling function was residential land, followed by commercial land (Fig 4). There were also a few education infilling patches surrounding the Nanjing High-tech Zone. Extension growth was characterized by the expansion of residential and commercial land in Hexi New Town, and manufacturing land in the Jiangning ETDZ and Banqiao New Town. Enclave was highlighted by manufacturing land expansion in the Nanjing ETDZ and the Jianging ETDZ, along with the expansion of education and residential lands in Xianlin University Town.

**Fig 5:**
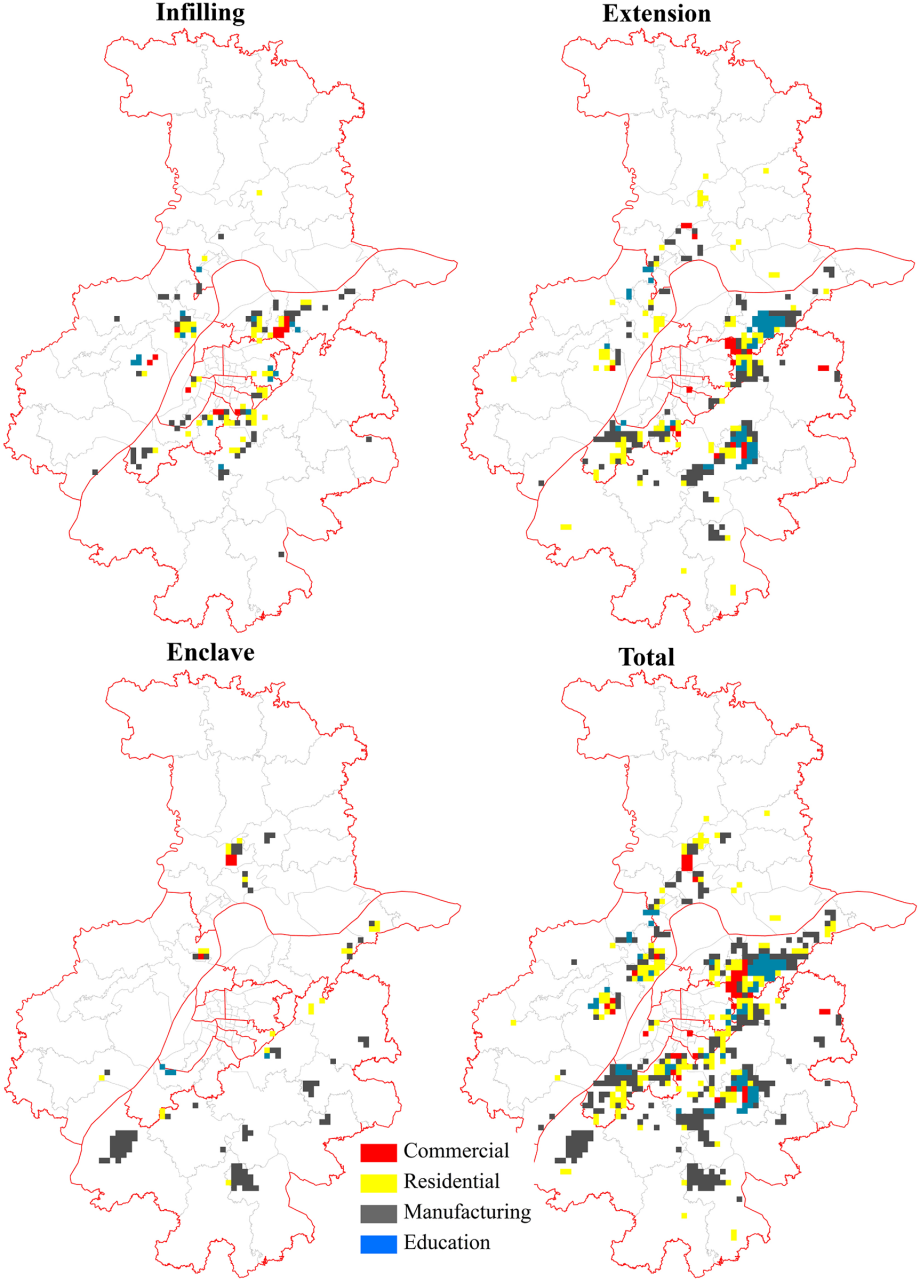
Main functions of newly grown urban patches from 2001 to 2007.

Moving into the new century, the distribution of newly-developed grids spread outwards under the polycentric urban form [38]. As Fig 5 indicated, infilling mainly occurred in three sub-centers and was characterized by a mix of residential and commercial lands. In addition, manufacturing land also infilled in levels of DZs. Extension was characterized by the expansion of education land in the two university towns of Xianlin and Jiangning, and by the expansion of manufacturing and residential lands for university-industry cooperation. Areas surrounding the Nanjing High-tech Zone and Banqiao New Town also witnessed rapid growth in manufacturing and residential lands. In contrast, enclave in this period was mainly located in Jiangning, surrounding the Binjiang DZ and Lukou International Airport, as well as other remote satellite towns.

**Fig 6:**
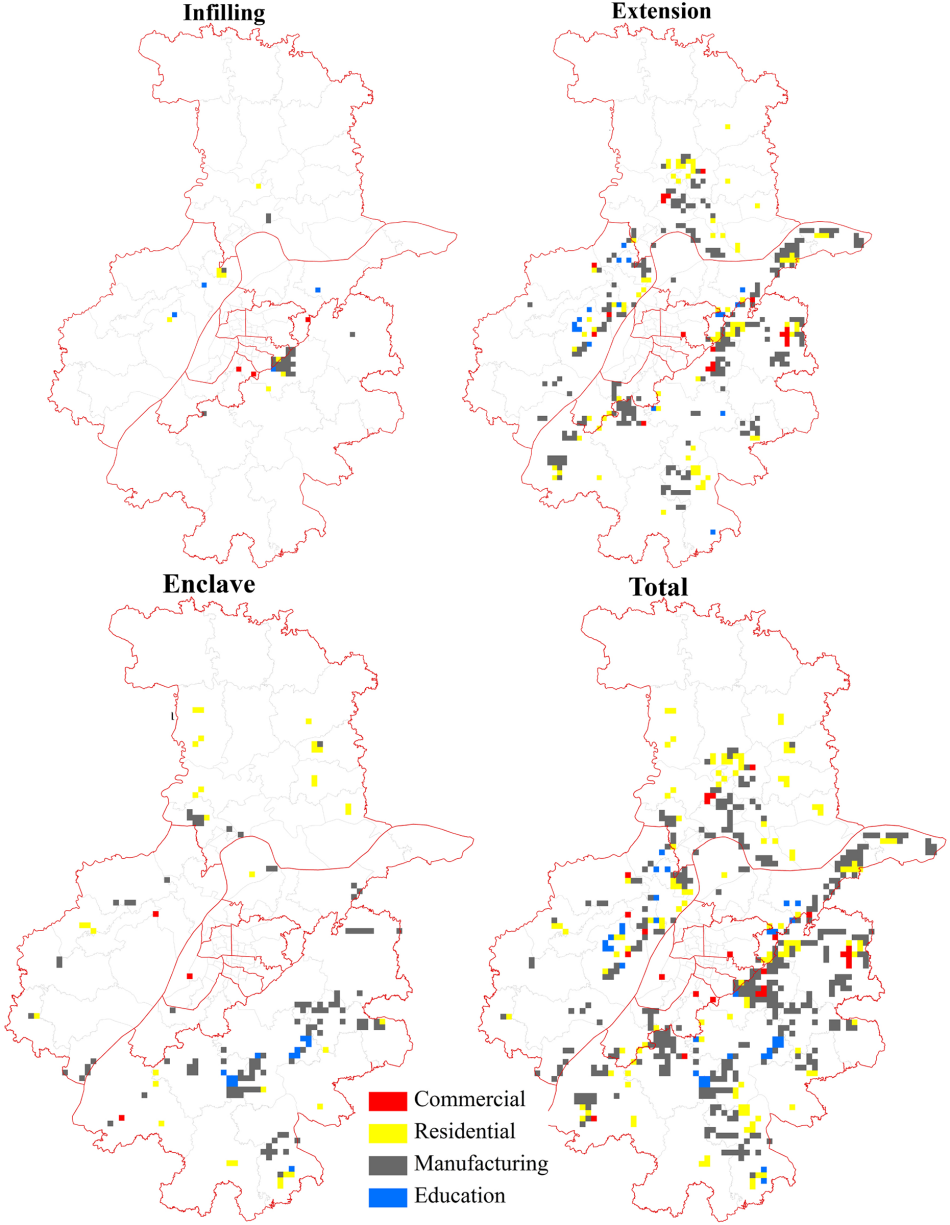
Main functions of newly grown urban patches from 2007 to 2013.

In the third period, newly-developed grids continued to spread outwards and were more dispersed than during the previous stage (Fig 6). Infilling decreased greatly and was characterized by the expansion of manufacturing land in the newly-established Qilin Technology and Innovation Park at the east of the city proper. Extension was dominated by manufacturing land at the fringe. Meanwhile, residential expansion occupied almost one-fourth of all the extension grids, which reflected a trend of job-housing balance in the process of industrial suburbanization [41]. Similar to the previous stage, enclave was still characterized by the rapid expansion of manufacturing land, mixed with small amounts of education in Jiangning. The difference between the two periods was that the reasons for enclave growth might have been the relocation of the old firms and the expansion of some universities (e.g., Southeast University, China Pharmaceutical University, Nanjing University of Aeronautics and Astronautics, Nanjing University of Posts and Telecommunications, and others) in the city proper [38]. With the increasing levels of urban-rural integration, some residential land enclaves were also created in remote towns [42].

To summarize (Table 4), on one hand, manufacturing expansion always dominated urban growth. This type of growth increased from appearing on 144 of 334 grids in the first period, to 266 of the 538 grids and 358 of the 542 grids in the last two periods, respectively. In contrast, residential expansion only increased from appearing on 112 of 334 grids in the first period, to 156 of the 538 grids in the second period, before decreasing to just 125 of the 542 grids in the third period. This decline was in line with the recent decay of the real estate market [43]. At the same time, commercial and education expansion occupied only from one-tenth to one-fifth of all the expansion grids in the study periods. These evolutionary processes are consistent with the findings of Gao; Wei; Chen and Yenneti [25] work on urban land expansion in the YRD.

**Table 4:** Main functions of newly developed urban land use patches in Nanjing.

Growth type	1995–2001					2001–2007					2007–2013				
	R	C	M	E	Total	R	C	M	E	Total	R	C	M	E	Total
**Infilling**	36	10	18	9	73	45	14	59	16	134	8	3	22	4	37
**Extension**	48	23	53	16	140	91	22	127	55	295	84	19	232	17	352
**Enclave**	28	7	73	13	121	20	5	80	4	109	33	3	104	13	153
**Total**	112	40	144	38	334	156	41	266	75	538	125	25	358	34	542

Note: R = Residential land, C = Commercial land, M = Manufacturing land, E = Education land.

Data in the table is the numbers of newly grown patches.

On the other hand, the leading growth type (manufacturing land expansion) was initially enclave but recently replaced by extension, together with infilling, in levels of the DZs and industrial parks. Residential land firstly expanded by types of infilling and extension in the late 1990s. Thereafter, the leading role of extension was highlighted, and enclave growth recently increased. Due to the relatively higher scale threshold, commercial land expansion was preferred to infilling and extension, rather than enclave growth. In addition, of the leading extension growth types, education land also expanded by the types of infilling and enclave. It is worth noting that infilling was an earlier phenomenon than enclave in terms of development stages, which is in line with the recent trend of extensive growth in China’s cities [44].

### Functional enhancing of the newly developed patches

In order to further understand the dynamics of the functional characteristics of urban land expansion in Nanjing, we further analyzed the functional evolution process of the newly-developed urban patches during the last two periods (Table 5). First, we selected 3×3 neighbors of all the expansion grids for the periods of 1995–2001 and 2001–2007. The functions of these neighbors in the following periods of 2001–2007 and 2007–2013 were detected. As illustrated in Table 5, all land use functions presented the trend of enhancing the process of expansion, excluding commercial land. In other words, urban land tended to strengthen the original function more so than that of transition.

**Table 5:** Functional evolution of newly developed urban patches in neighboring periods.

1995–2001	2001–2007					2001–2007	2007–2013				
	R	C	M	E	Total		R	C	M	E	Total
**R**	96	26	45	24	191	**R**	72	7	87	12	178
**C**	20	18	22	7	67	**C**	13	11	14	2	40
**M**	64	11	112	19	206	**M**	38	10	192	13	253
**E**	28	7	25	47	107	**E**	8	4	22	20	54
**Total**	208	62	204	97	571	**Total**	131	32	315	47	525

Note: R = Residential land, C = Commercial land, M = Manufacturing land, E = Education land.

Data in the table is the numbers of newly grown patches.

As 64 and 38 grids surrounding previously-designated manufacturing land were developed into residential land in 2001–2007 and 2007–2013 respectively, it can be noted that manufacturing development can largely promote the expansion of residential land. While, expansions of manufacturing and education land have largely promoted each other, thereby confirming the trend of university-industry cooperation over the last decade. At the same time, residential land expansion has naturally promoted the growth of commercial, education, and manufacturing. These findings reinforce the previous work of Feng; Dijst; Wissink and Prillwitz [45], who employed an analysis of mode choice for commuting and shopping-leisure trips in Nanjing.

## Conclusions

The spatiotemporal patterns and dynamics of urban growth had been a consistently discussed topic in urban studies. With increasing acceptance of the concept of smart growth and sustainable development, scholars had paid renewed attention to urban expansion, especially in Chinese cities [46]. However, there is a paucity of research on integrating different scales of analyses on urban growth types and functions remains largely undone. Using Nanjing as a case, we quantified and highlighted the spatiotemporal patterns, growth types, and functional evolution. This could provide a case to further understand urban growth in fast-growing second tier cities in China, which is both timely and necessary.

The research of our findings reveal that due to topographic constraints and low economic and population growth rates, urban land expansion in Nanjing was relatively slow prior to 1995, as was very common in the Yangtze River Basin [47]. Thereafter, expansion expedited with the development of levels of development zones (DZs) and new towns in the late 1990s. This expansion echoed the notion of development zone fever and the investment-driven expansion model in China [48]. At the onset of the 21^st^ century, urban land expansion further accelerated with the initiation of official spatial planning, which has been presented in other case studies in China [31, 47]. The growth area increased annually, even though the annual growth rate declined somewhat when the city form shifted from a compact to a polycentric form in recent years.

Combining the common edge method at a patch scale and the convex hull method at a city scale, this study also developed a new approach, which has rarely been applied in previous studies. That method is a comprehensive matrix analysis. Based on the integration of methods at double-scales, our research casts a new light on the spatiotemporal patterns of three growth types. Also, at this comprehensive scale, infilling growth dominated urban land expansion in the early stage of urbanization, while extension and enclave growth sharply increased when the city became more compact. When the polycentric pattern formed, infilling rebounded with the continuous extension of the sub-centers and enclaves of satellite towns. In reciprocation, the urban growth and land sprawls continue.

Our findings also imply that both the spatial patterns and functions of land expansion varied depending on growth types. Infilling growth was initially dominated by residential land centered on the original city proper, and latterly by a mix of residential and manufacturing land at the fringe, as was recently characterized by the manufacturing expansion in subcenters. Extension growth was characterized by a mix of residential and industrial land growth at the city’s fringes, while growth has shifted towards using land for manufacturing purposes in the subcenters, while retaining a certain scale of residential expansion around the previously-expanded patches. These growth patterns are in line with the establishment of development zones and new towns. Enclave growth was consistently dominated by and for manufacturing purposes in pre-urban areas located at a distance of from several to tens of kilometers from the city center or subcenters, in conjunction with land expansion for education purposes in university towns and for residential purposes in remote towns.

With respect to enhancing functional characteristics, the newly-developed urban land was characterized by the strengthening of its original function, excluding commercial land, reflecting a trend of clustering manufacturing and residential land, as well as the service market limits. We also found some evidence of mutual promotion between different functions during the expansion process. Residential land preferred to expand by surrounding manufacturing land, because of the requirement for a jobs-housing balance. Increasing education land more or less promoted the expansion of manufacturing land, reflecting the university-industry cooperation. Naturally, the expansion of residential land also attracted considerable commercial and education land growth in the early period. Consequently, these mutual promotions led to the formation of concentric spatial patterns, represented as the growth of commercial, residential, and industrial land from inner to suburban areas.

Finally, the findings of this study can be strengthened by more fully taking the driving forces underlying the three specific growth types into account. For instance, recent research has tried to address the endogenous effects of development policies and economic restructuring on urban land expansion [34]. However, it is largely based on qualitative theoretical analysis. From a methodology perspective, both a spatial regime model and geographically and temporally weighted regression [25] can be employed to investigate the heterogeneity in dynamics of the three growth types. Furthermore, the inner restructuring process of urban renewal and regeneration could be discussed as a supplemental study for the current hot topic of outward expansion [49, 50]. From a method perspective, the new integrated method developed in this article has limitation and difficulties when applied to other cases. One is that the images cannot specifically reflect the real trajectories of urban land sprawl; another is the boundary determination of the existing pre-growth urban areas.

## Supporting Information

S1 Fig The visual illustrations to describe all possible scenarios. (DOCX)
