# Supplementary material for: Growth Type and Functional Trajectories: An Empirical Study of Urban Expansion in Nanjing, China
Source: PLoS One. 2016 Feb 4;11(2):e0148389. doi: 10.1371/journal.pone.0148389 (PMC4742073; doi:10.1371/journal.pone.0148389)
Supplement: S1 Fig — (DOCX) [file pone.0148389.s001.docx]

**Supporting Information**

**S1 Fig. The visual illustrations to describe all possible scenarios.**


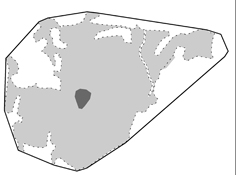

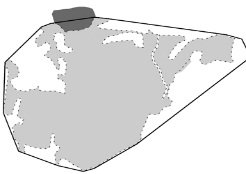

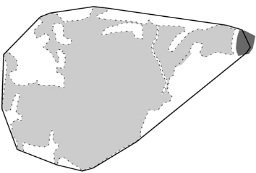


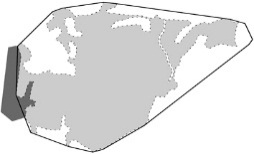

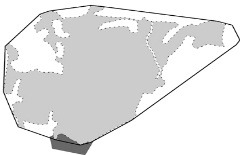

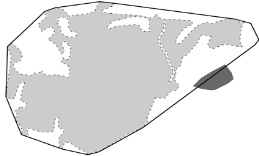


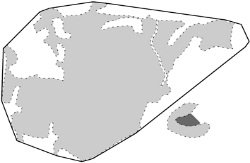

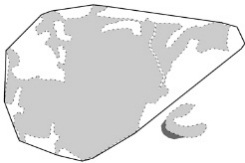

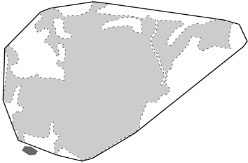


Note: The black line denotes the convex hull of major urban area; the light polygon denotes the pre-growth patches; the dark one denotes the newly expanded.

The order of these figures are same with Table 1.
